# Endoplasmic reticulum chaperone prolyl 4-hydroxylase, beta polypeptide (P4HB) promotes malignant phenotypes in glioma via MAPK signaling

**DOI:** 10.18632/oncotarget.18026

**Published:** 2017-05-19

**Authors:** Stella Sun, Karrie M.Y. Kiang, Amy S.W. Ho, Derek Lee, Ming-Wai Poon, Fei-Fan Xu, Jenny K.S. Pu, Amanda N.C. Kan, Nikki P.Y. Lee, Xiao-Bing Liu, Kwan Man, Philip J.R. Day, Wai-Man Lui, Ching-Fai Fung, Gilberto K.K. Leung

**Affiliations:** ^1^ Department of Surgery, Li Ka Shing Faculty of Medicine, The University of Hong Kong, Queen Mary Hospital, Pokfulam, Hong Kong; ^2^ Department of Pathology and Clinical Biochemistry, Queen Mary Hospital, Pokfulam, Hong Kong; ^3^ The Manchester Institute of Biotechnology, Faculty of Biology, Medicine and Health Sciences, University of Manchester, Manchester, United Kingdom

**Keywords:** P4HB, angiogenesis, glioma, invasion, MAPK signaling

## Abstract

Endoplasmic reticulum (ER) chaperone Prolyl 4-hydroxylase, beta polypeptide (P4HB) has previously been identified as a novel target for chemoresistance in glioblastoma multiforme (GBM). Yet its functional roles in glioma carcinogenesis remain elusive. In clinical analysis using human glioma specimens and Gene Expression Omnibus (GEO) profiles, we found that aberrant expression of P4HB was correlated with high-grade malignancy and an angiogenic phenotype in glioma. Furthermore, P4HB upregulation conferred malignant characteristics including proliferation, invasion, migration and angiogenesis *in vitro*, and increased tumor growth *in vivo* via the mitogen-activated protein kinase (MAPK) signaling pathway. Pathway analysis suggested genetic and pharmacologic inhibition of P4HB suppressed MAPK expression and its downstream targets were involved in angiogenesis and invasion. This is the first study that demonstrates the oncogenic roles of P4HB and its underlying mechanism in glioma. Since tumor invasion and Vascularisation are typical hallmarks in malignant glioma, our findings uncover a promising anti-glioma mechanism through P4HB-mediated retardation of MAPK signal transduction.

## INTRODUCTION

Glioblastoma multiforme (GBM) is characterised by its highly infiltrative and proliferative invasion of surrounding tissues, which makes it one of the most lethal types of primary brain tumours [1, 2]. Despite multimodal treatments involving surgical resection, chemotherapy and radiotherapy, patients with GBM have life expectancies of only around 15 months after diagnosis [3]. One major reason for treatment failure is GBM's aggressive behaviour that invade surrounding tissues, which precludes complete tumour excision [4, 5]. Exploiting the signaling mechanisms that drive tumour progression and invasion could be important for the development of therapeutics for this deadly disease.

Endoplasmic reticulum (ER) is the major site for protein synthesis, and the ER chaperone functions to ensure proper folding of newly synthesized proteins. With the increment of protein synthesis in proliferating cancer cells, the activities of ER chaperones experience high demand that might lead to the activation of unfolded protein response (UPR) in cancer cells [6, 7]. While this class of proteins was traditionally thought to be primarily responsible for protein folding and post-translational modification within the ER, they are now also found to be critical in regulating proliferation, apoptosis and immunity [6, 8]. Moreover, due to the high interconnectivity of the ER with other cellular compartments, the functions of ER chaperones may also extend beyond the ER by regulating pro-oncogenic and pro-survival signals [9, 10]. Therefore, perturbation of ER homeostasis could have critical roles in Tumourigenesis, and targeting ER chaperones represents a novel direction for developing anti-tumor therapy.

Prolyl 4-hydroxylase, beta polypeptide (P4HB) is one of the major chaperone proteins [11]. Like other ER chaperones, P4HB, is also located on the cell surface, with activities distinct from those in the ER [12, 13]. These findings expanded the paradigm of P4HB functions, and implied a high versatility of this protein in different biological processes. Elevated levels of P4HB may confer tolerance against extracellular stresses such as hypoxia and ischemia [14]. Its expression following serial transplantations of clinical glioblastoma xenografts in the brains of immunodeficient rats also showed correlations with invasive properties [15].

Recently, we showed that upregulation of P4HB was associated with temozolomide (TMZ) resistance in malignant glioma, and that its inhibition may sensitize chemoresistant glioma to TMZ treatment [16]. This study aimed at delineating the oncogenic role of P4HB in malignant glioma. Our work provided novel findings that aberrant expression of P4HB promotes tumour invasion, angiogenesis and growth via the MAPK signaling pathways. These multifunctional roles implicate its clinical significance in cancer. Targeting P4HB with consequential blockage of these pathways may provide an alternative treatment approach for GBM.

## RESULTS

### Upregulated P4HB expression is associated with high-grade human glioma

**Figure 1:**
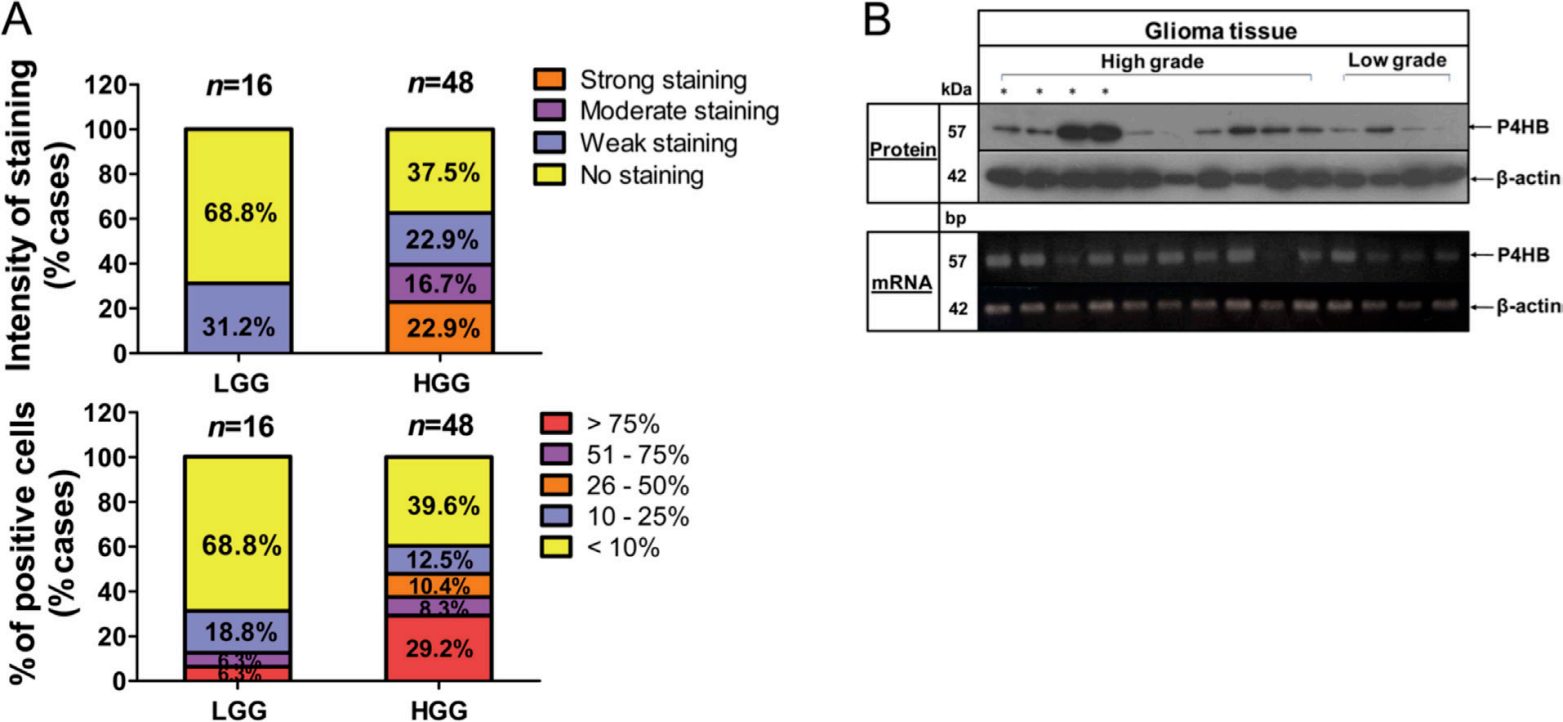
Upregulation of P4HB is associated with high grade glioma (**A**) P4HB immunostaining was assessed on a patient set of 64 cases. Scoring system was calculated based on intensity of staining (none, weak, moderate and strong; top) and percentage of positively stained cells (< 10%, 10%–25% 26%–50%, 51%–75% and > 75%; bottom) in low-grade glioma (LGG) and high-grade glioma (HGG) specimens. (**B**) Representative images demonstrated P4HB protein (upper panel) and gene expression (lower panel) in low-grade and high-grade glioma specimens as demonstrated by western blotting and RT-PCR, respectively. β-Actin was used as an internal loading control.

We first assessed cytoplasmic P4HB expression in 64 human glioma specimens based on the staining intensities and percentage of positively stained cells. Levels of intensity varied among different cancer grades, in that P4HB was detectable in 30/48 (62.5%) high-grade, but only 5/16 (31.2%) in low-grade gliomas. Only 12.6% of low-grade glioma cases demonstrated more than 25% of P4HB-stained cells, which was around 30% relative to high-grade glioma (47.9% cases), suggesting that P4HB expression was correlated with glioma malignancy grades (Figure 1A). High P4HB expression in high grade gliomas was further validated by western blot and end-point PCR analysis at the protein and mRNA levels (Figure 1B).

### P4HB expression is correlated with increased angiogenesis

**Figure 2:**
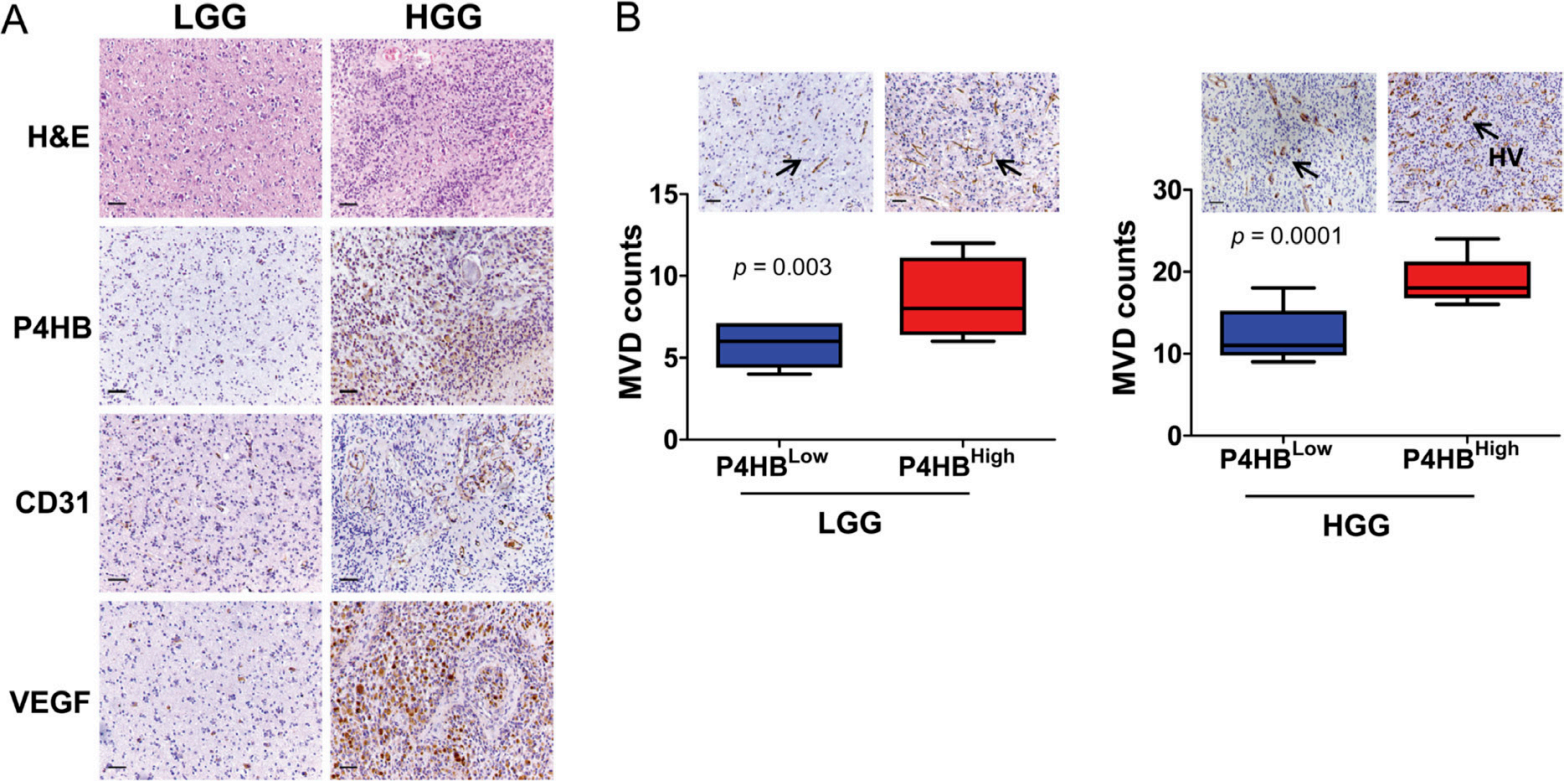
P4HB plays roles in glioma angiogenesis (**A**) Immunostaining of high grade glioma (HGG) and low grade glioma (LGG) specimens showed that HGG was associated with upregulation of P4HB, CD31 and VEGF expressions. (**B**) Increase in microvessel density (by CD31-positive staining) was associated with upregulation of P4HB (P4HB^High^) in both HGG in both HGG (*n* = 48; ***p = *0.0001*) and LGG (*n* = 16; **p = *0.003*). The microvessels in LGG were pericytes-liked with capillary phenotype (black arrows) whereas those in HGG showed hypervascularity (HV) of enlarged, branched and disorganized vessel structures (black arrows). Microvessels were found to be most abundance in HGG with strong P4HB expression (Scale bars: 50 μm).

In addition to clinical grading (*p* = 0.013), P4HB expression in malignant glioma was also found to be significantly correlated with angiogenesis as indicated by CD31 expression (*p* = 0.009), and a positive association with VEGF (*p* = 0.07) (Table 1). Microvessels (arterioles, venules, and capillaries) densities and VEGF staining intensities were significantly stronger in high-grade (high P4HB expression) when compared to the low-grade glioma (low P4HB expression) (Figure 2A). Distinctive vessel staining patterns, revealed by CD31 staining, were observed in a grade-dependent manner with differential P4HB expressions. In low-grade glioma with low P4HB expression, microvessels were predominantly pericyte-liked with a capillary phenotype, whereas in high-grade glioma there was hypervascularity with enlarged, branched and disorganized vessel structures. Microvessel density (MVD) correlated positively with P4HB in both low-grade (*p* = 0.003) and high-grade gliomas (*p* = 0.0001) (Figure 2B).

**Table 1:** Correlation between P4HB protein expression and clinical/angiogenic variables

Variables	No. of cases (*n*, %)	P4HB protein expression		*P* value
		Low (*n*, %)	High (*n*, %)	
**Gender**				
Male	37 (57.8)	23 (62.2)	14 (37.8)	0.275
Female	27 (42.2)	21 (77.8)	6 (22.2)	
**Age**				
< 49.2	32 (50.0)	26 (81.3)	6 (18.8)	0.058
> 49.2	32 (50.0)	18 (56.3)	14 (43.8)	
**Grading**				
Low grade	16 (25.0)	15 (93.8)	1 (6.3)	**0.013†**
High grade	48 (75.0)	29 (60.4)	19 (39.6)	
**MGMTϗ**				
Unmethylated	21 (47.7)	13 (61.9)	8(38.1)	0.752
Methylated	23 (52.3)	16 (69.6)	7 (30.4)	
**VEGFϗ**				
Low	23 (41.1)	14 (60.9)	9 (39.1)	0.070
High	33 (58.9)	12 (36.4)	21 (63.6)	
**CD31ϗ**				
Low	24 (42.9)	16 (66.7)	8 (33.3)	**0.009†**
High	32 (57.1)	10 (31.3)	22 (68.8)	

^†^Statistically significant

^ϗ^Partial data are not available, and statistics were based on available data.

### Differential gene expression and biological processes associated with P4HB gene expression

**Figure 3:**
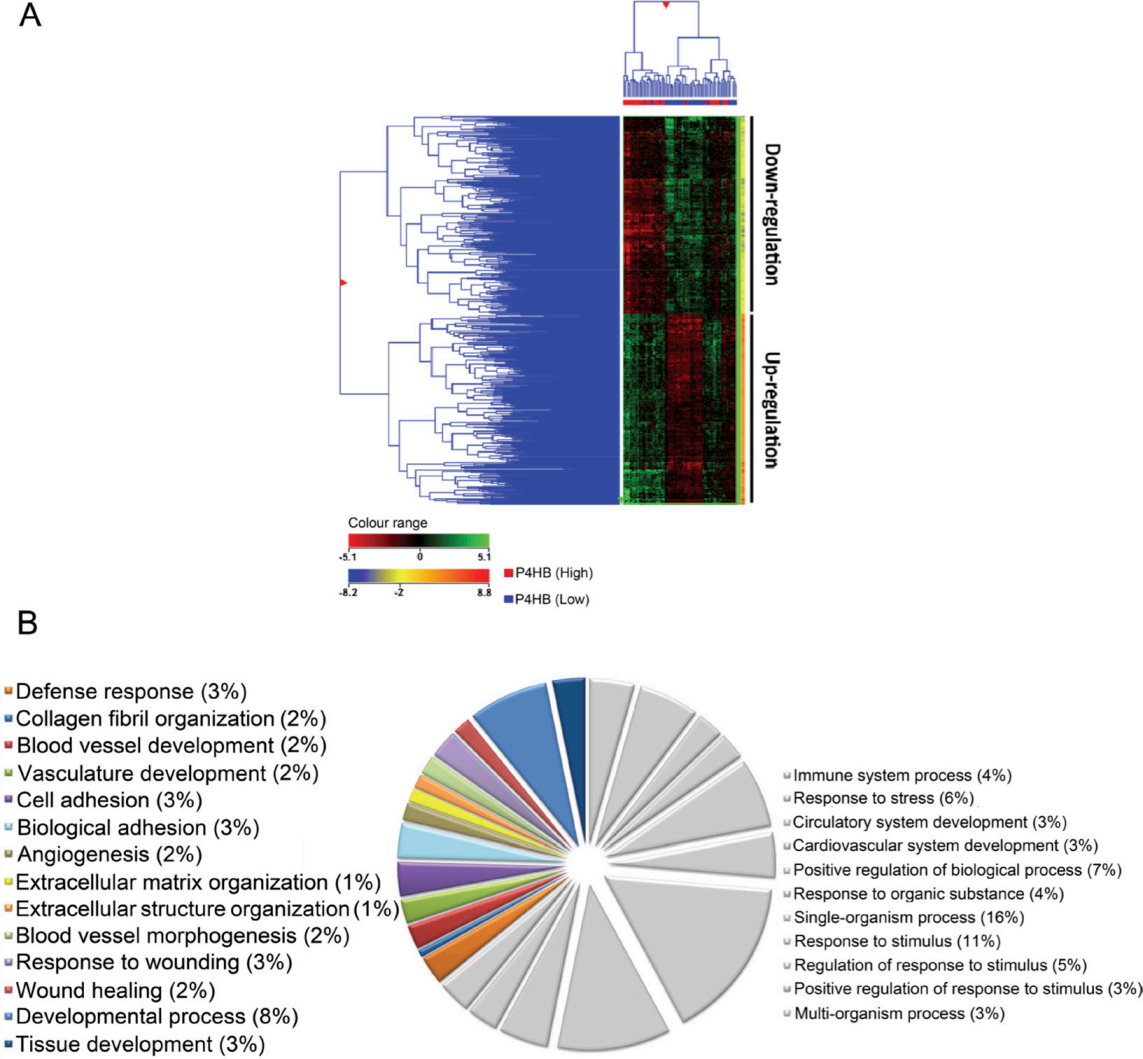
Unique gene expression signature was identified from samples of P4HB^Low^ and P4HB^High^ glioma specimens (*n* = 73) (**A**) By hierarchical clustering analysis, (2-fold cutoff; *p* ≤ *0.001*), a differential gene expression pattern was observed between P4HB^High^ and P4HB^Low^ groups. (**B**) Graphical illustration of the most representative gene ontology (GO) identifiers within the GO category ‘biological processes’, including developmental process, tissue development, invasion purposes, angiogenesis and wound healing. GO analysis showed significant associations (*p* ≤ *0.00001*) of genes involved in tumor progression and regulation within the P4HB^High^ group when compared with the P4HB^Low^ group.

To interrogate the potential mechanisms underlying P4HB's action in the present context, we studied the gene expression profiles of 73 patient-derived tissues obtained from a publicly available database GSE16011. Hierarchical clustering graphically displayed distinct differential gene expression patterns between low- and high- P4HB groups (Figure 3A). A total of 2227 differentially expressed genes were identified, of which 756 were showed two-fold or more differences.

Gene ontology (GO) enrichment analysis confirmed that most up-regulated genes were featured with aspect to blood vessel, vasculature and tissue development. Others were involved in cell adhesion, extracellular matrix organization and wound healing (Figure 3B). The findings suggested that P4HB may regulate a wide range of gene functions but predominantly in angiogenesis and Tumour regulation. Using GeneSpring^™^, we have identified several canonical signaling pathways that are significantly correlated with high P4HB expression (*p* < 0.001) (Supplementary Table 1). Of interest is the involvement of MAPK signaling pathway together with focal adhesion and angiogenesis, which based on the degrees of correlation, our previous findings and literature reviews, were subject to further investigations in this study.

### Involvement of MAPK signaling in *P4HB*-dependent oncogenic effects

**Figure 4:**
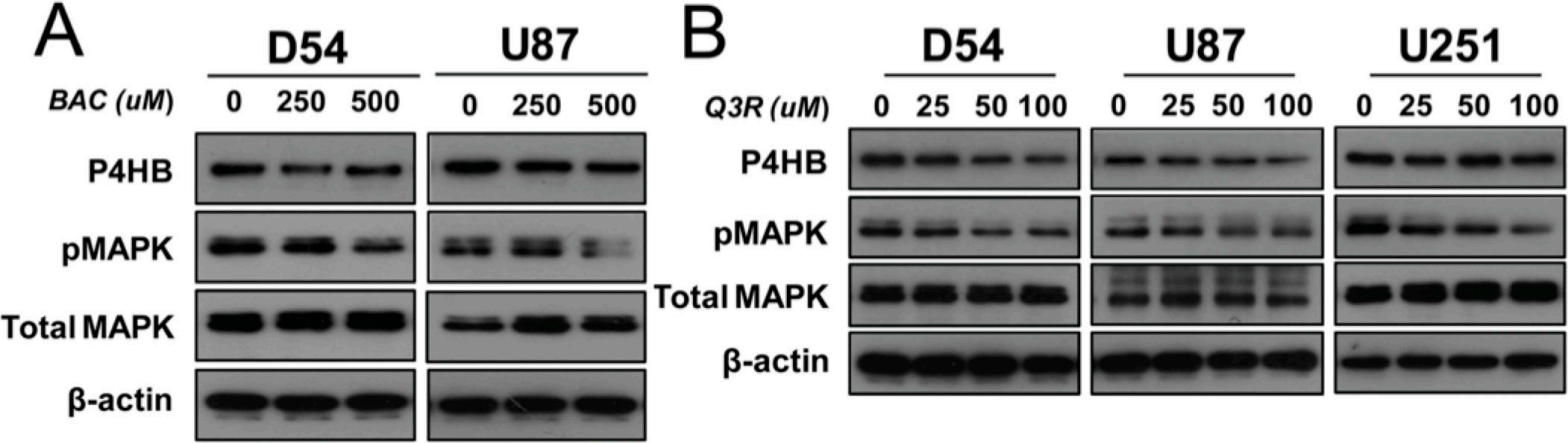
Involvement of MAPK signaling in P4HB-dependent oncogenic effects (**A**) Increasing concentration (0, 250, 500 μM) of bacitracin, a P4HB inhibitor (24-hour treatment), resulted in inhibition of MAPK phosphorylation in D54 and U87 glioma cells. (**B**) Increasing concentration (0, 25, 50, 100 μM)) of Q3R, another potent selective inhibitor of P4HB (48 hour treatment), suppressed MAPK phosphorylation in D54, U87 and U251 glioma lines. β-Actin was used as an internal loading control.

To study the role of MAPK, D54 and U87 cells were treated with increasing concentrations (0, 250 and 500 μM) of bacitracin (BAC), a P4HB inhibitor. A dose-dependent inhibition of MAPK phosphorylation was observed after 24 hour of treatment (Figure 4A). Treatment with quercetin-3-rutinoside (Q3R), another potent selective inhibitor of P4HB, at increasing concentrations (0, 25, 50, 100μΜ) for 48 hours similarly suppressed MAPK phosphorylation in D54, U87 and U251 cells (Figure 4B). The findings suggested that P4HB-dependent oncogenic activities are associated with MAPK signaling.

### Over-expression of P4HB promotes cell proliferation, migration, invasion and tube formation ability *in vitro*

**Figure 5:**
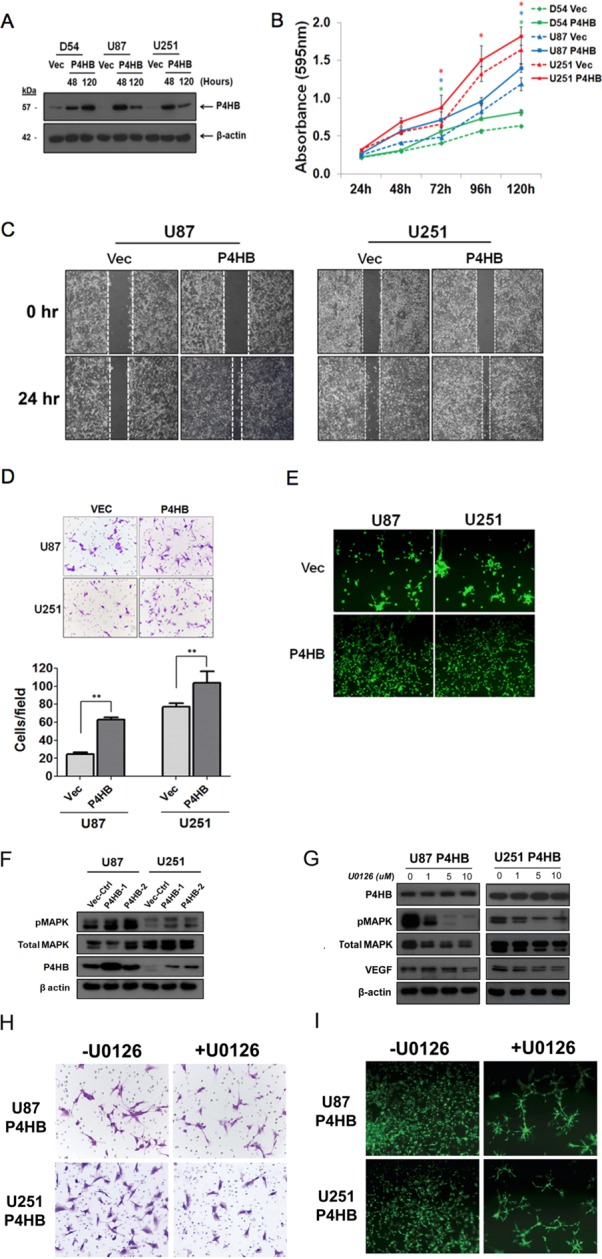
Transient over-expression of P4HB promoted glioma cell proliferation, migration, invasion and tube formation ability *in vitro* (**A**) Western blot analysis showed upregulated expression of P4HB post-transfection. (**B**) MTT assay was performed on cells with transient P4HB over-expression (D54 P4HB, U87 P4HB and U251 P4HB) and empty vector controls (D54 Vec, U87 Vec and U251 Vec). After 5 days incubation, cells over-expressing P4HB showed higher proliferative rates than controls. (**C**) Migration assay showed greater motility of U87 P4HB and U251 P4HB than their respective vector controls (U87 Vec and U251 Vec). (**D**) Matrigel cell invasion assay similarly showed greater invasiveness in U87 and U251 cells with P4HB over-expression (** *p* < *0.05*). (**E**) Angiogenesis, as measured by tube formation ability, was again higher in P4HB overexpressing cells (U87 and U251). (**F**) Western blot analysis revealed that P4HB over-expression was associated with increased MAPK phosphorylation. (**G**) Suppression of MAPK activities by U0126 reduced VEGF expression in P4HB over-expressing cells, while P4HB expression level was unaffected. (**H**) Representative pictures from three independent assays showed decreased cell invasion abilities of U87 P4HB and U251 P4HB cells after treatment with U0126. (**I**) U0126 pretreatment also inhibited tube formation in these cells after 24 h (Magnification: ×200).

Having found that P4HB expression was associated with glioma malignant phenotypes, we then performed gain-of-function *in vitro* assays. Figure 5A illustrates that GBM cells with P4HB over-expression were successfully established in D54, U87 and U251 cells (D54-P4HB, U87-P4HB and U251-P4HB). When compared with the vectors controls (D54-Vec, U87-Vec and U251-Vec), cells with P4HB over-expression exhibited significantly (p < 0.05) higher proliferative rates on MTT assay at 120 hour (Figure 5B). GBM cells with P4HB over-expression also exhibited greater migration abilities at 24 hour than vector controls (Figure 5C). A similar trend was observed on matrigel invasion assay, with U87-P4HB and U251-P4HB cells showing greater invasion ability at 24 hours when compared to their respective vector controls (*p* < 0.001) (Figure 5D). Angiogenic ability was also measured on tube formation assay, parental cells showed no branching and tube network formation while GBM cells with P4HB over-expression aligned to form branch-like and net like (Figure 5E).

To further examine the involvement of MAPK activities in P4HB-dependent oncogenic effects, MAPK phosphorylation was shown to be enhanced in P4HB over-expressing U87 and U251 cells (U87 P4HB-1, U87 P4HB-2, U251 P4HB-1 and U251 P4HB-2) when compared with the vector controls (U87 Vec-Ctrl and U251 Vec-Ctrl) (Figure 5F). Suppression of MAPK activities by using U0126, at 0, 1, 5, 10 μM for 2 hours in P4HB over-expressing U87 and U251 cells (U87 P4HB and U251 P4HB) was found to reduce VEGF expression without affecting P4HB level (Figure 5G). The invasive and angiogenic abilities of U87 P4HB and U251 P4HB cells were affected phenotypically after the reduction of MAPK activity (Figure 5H and 5I). These findings lend further support to the suggestion that the oncogenic activities of P4HB are, at least partially, mediated by downstream MAPK signaling.

### P4HB enhances Tumourigenicity in orthotopic brain tumor grafts

**Figure 6:**
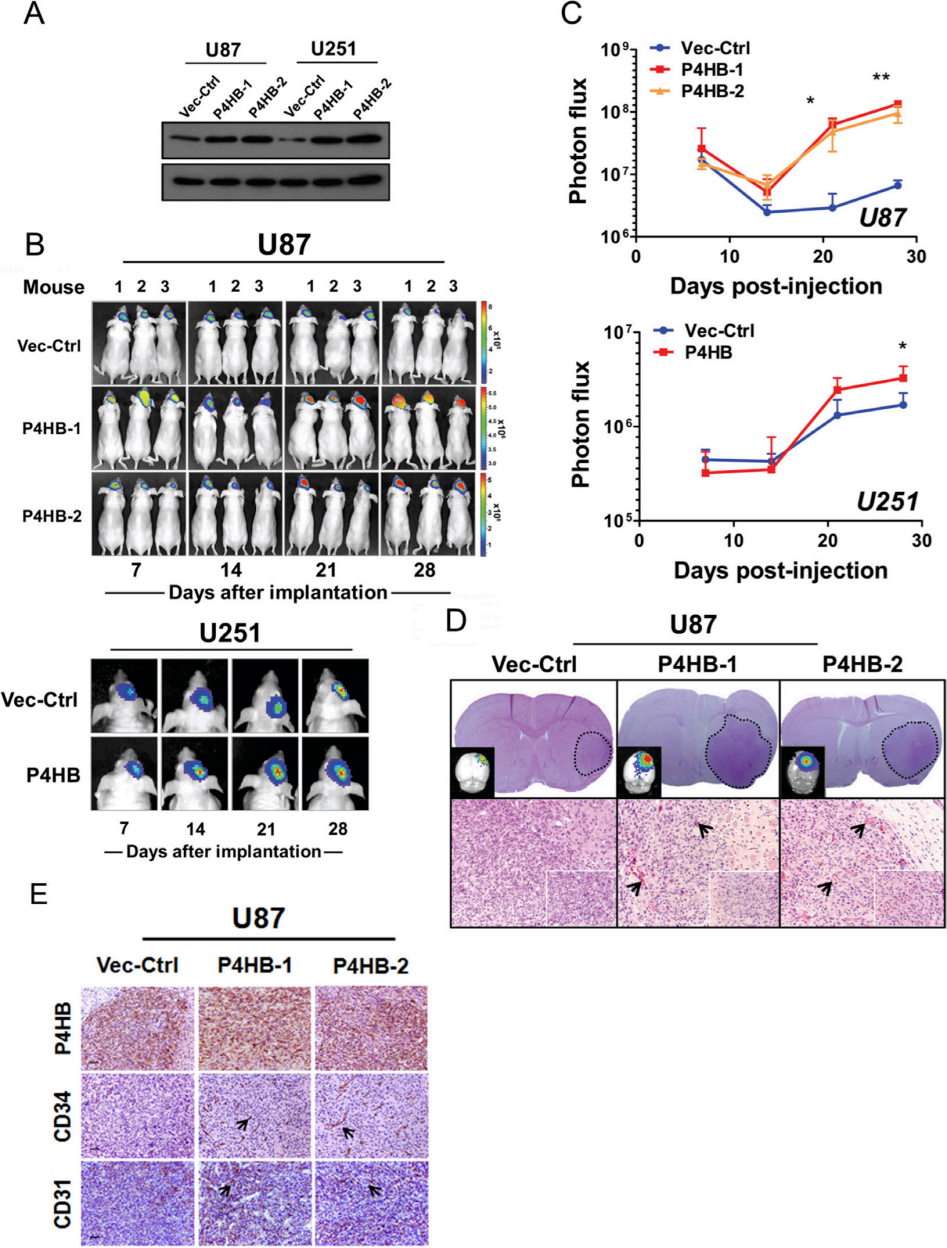
Stable P4HB over-expression was associated with increased tumour growth *in vivo* in an orthotropic glioma model (**A**) Western blot analysis confirmed stable P4HB over-expression in transfected cells (U87 P4HB-1 and U87 P4HB-2; U251 P4HB-1 and U251 P4HB-2). Stable clones of empty vector (D54 Vec-Ctrl and U87 Vec-Ctrl) were used as controls. (**B**) Quantitative BLI was performed for 28 days starting at one week post-transplantation. Representative images demonstrated luciferase activities in tumor bearing mice at indicated time-points (Day 7, 14, 21 and 28) post-transplantation. Heat-map scale bar represents photon emission (Units = photons/s/cm2/steradian). (**C**) U87 P4HB-1, U87 P4HB-2 and U251 P4HB cells showed significant increase in bioluminescence activities when compared with controls at 28 days post-injection. (*p < *0.05*). (**D**) Coronal section of U87-tumor-bearing mice brain showing a well circumscribed tumor within the cerebral hemisphere (dotted line; upper panel). Detection of tumor in *ex vivo* grafts was confirmed using luminescence (top left corner). H&E staining of representative P4HB-over-expressing U87 tumors showed intense tumor vessel densities when compared with controls. (Original magnification: ×100; ×400 (insets). (**E**) Immunohistochemical analysis showed expression correlations between P4HB and CD34/CD31 in U87 P4HB-1 and P4HB-2 *ex vivo* grafts. Representative features are indicated by black arrows (Scale bars: 50 μm).

Lastly, we studied the effect of stable P4HB over-expression (U87 P4HB-1, U87 P4HB-2 and U251 P4HB) on tumourigenicity *in vivo* using an orthotopic xenograft model (Figure 6A). At 14 day post-implantation, stable P4HB cells (U87 P4HB-1, U87 P4HB-2 and U251 P4HB) exhibited significantly greater exponential growth when compared with vector control (U87 Vec-Ctrl and U251 Vec-Ctrl) (*p < 0.05*) (Figure 6B). At 28 day, the mean tumor luciferase radiance (in photons/sec/cm^2^/sr) for U87 implants were 6.62 × 10^6^ (Vec-Ctrl), 1.34 × 10^8^ (P4HB-1) and 1.28 × 10^8^ (P4HB-2). For U251 implants, the luciferase radiances were 1.70 × 10^6^ (Vec-Ctrl) and 3.27 × 10^6^ (P4HB) (Figure 6C). *Ex vivo* histologic examination of the tumor grafts by H&E showed increased vascularity (Figure 6D) as well as increased P4HB, CD31 and CD34 staining in P4HB over-expressing cells (Figure 6E). The overall findings suggest that upregulated P4HB expression is associated with growth advantage, possibly through increased angiogenesis.

## DISCUSSION

Neoplastic progression is a multistep process of genetic mutations and adaptive responses that can over-ride growth arrest or senescence controls [17]. Adaptive endoplasmic reticulum stress response (ERSR) due to chronic ER stress (e.g., caused by hypoxia and glucose deprivation) is a common feature in rapidly proliferating cancer cells [18], and may protect tumor cells against further exogenous insults such as chemotherapeutics [19, 20]. ERSR is carried out by chaperones whose main functions are to facilitate protein folding and the eradication of malformed proteins [21], but recent discoveries indicate that chaperones may also affect tumor cell growth and signaling [22].

Glioma cells are in a constant state of low grade ERSR that possibly contributes to their resistance to chemo-irradiation [21]. The most abundant and well-characterised ER chaperone, GRP78 has been identified to be critical for tumourigenesis and therapeutic resistance [6, 23], exhibiting elevated expression in many cancers, including glioma [24]. Cancer cells with constitutively upregulated GRP78 could tolerate chemotherapeutic agents through the suppression of pro-apoptotic pathways [25]. For oncogenesis, GRP78 could induce the activation of AKT signaling during leukemogenesis and prostate tumorigenesis [26–28]. A conditional heterozygous deletion of GRP78 may reduce tumour angiogenesis and metastatic growth in tumor endothelial cells but not in normal tissue [29]. Other chaperones such as GRP 94, calreticulin (CRT) and protein disulfide isomerase (PDI) are similarly implicated [30–32].

Our group has previously identified another ER chaperone protein, P4HB, to be associated with TMZ resistance in malignant glioma [33]. Down-regulation of P4HB enhanced chemosensitivity via the ERSR signaling pathway both *in vitro* and *in vivo*, implying that it is a potential target for chemoresistant GBM [16]. The P4HB family also has neuroprotective actions in various neurodegenerative diseases and cerebral ischemia [34–36]. As such, it is conceivable, though unproven, that high P4HB expression in brain tumours may also exert impact on disease progression and clinical outcome. Goplen *et al*. were first to identify the role of P4HB in cancer invasion but no functional studies or clinical validation were reported [15]. Other investigators have also described its protective role in post-injury response and anti-tumor immunity, its role in glioma tumourigenesis has been elusive [37, 38]. The present study is the first to describe the oncogenic role of P4HB in GBM and the mechanisms underlying its contribution to glioma progression.

MAPK signaling is one of the main EGFR downstream pathways that are known to be critical for glioma tumourigenesis [39, 40]. MAPK signaling are evolutionarily important linkages to the machinery that controls fundamental cellular processes such as proliferation, migration and apoptosis [41]. Abnormalities in MAPK signaling play a critical role in the development and progression of cancer [42]. Recently, STL1, the co-chaperone stress-in-double protein 1, is reported to promote glioma proliferation through MAPK pathways, suggests a possible link between MAPK and chaperone activities in the development in glioma [43].

Interestingly, we found that up-regulation of P4HB was significantly associated with MAPK activation signaling and the critical downstream processes of angiogenesis and invasion. We also delineated the influences of P4HB on the regulations of VEGF. By adversely perturbing P4HB *in vitro*, MAPK and VEGF expressions decreased; contrawise, P4HB over-expression had the opposite effects. To further verify our findings, we suppressed P4HB expression pharmaceutically. BAC is widely used as a P4HB inhibitor of uncertain specificity of action [44], whereas Q3R has more selective mode of action [13]. We observed that both inhibitors could suppress MAPK phosphorylation, suggesting that P4HB may regulate MAPK signaling. Conversely, inhibiting MAPK pathway with U0126 abrogated the induction of VEGF, tumor invasion and angiogenesis, without alterations in P4HB protein levels. The overall findings suggested that MAPK signaling was downstream to P4HB.

## MATERIALS AND METHODS

### Human glioma specimens and cell lines

A total of 64 specimens (mean age: 49.48 ± 13.80 years) obtained from 2002 to 2010 were retrieved from our institution's tissue bank and comprised 16 low-grade gliomas (WHO grade II), 12 anaplastic gliomas (WHO grade III) and 36 GBM (WHO grade IV) [2]. The diagnosis of malignant glioma was made radiologically via magnetic resonance imaging (MRI) before surgery, and confirmed histologically by a certified pathologist. The study was approved by the Institutional Review Board of our institution, and all tissues were collected with signed informed consent from patients.

Human GBM cell lines D54-MG and U87-MG were obtained and were described in our previous study [33]. U251-MG cell line was obtained from the American Type Culture Collection (ATCC; Manassas, VA, USA). D54-MG was cultured in Dulbecco's modified Eagle's medium (DMEM)/F12 (1:1), whilst U87-MG and U251-MG were cultured in minimum essential medium (MEM)-α (GIBCO^®^; Life Technologies, Inc., Carlsbad, CA, USA) at 37°C in 5% CO_2_, 90% relative humidity. All media were supplemented with 10% heat-inactivated fetal bovine serum (FBS) (GIBCO^®^), 100 IU/ml penicillin and 100 μg/ml streptomycin (GIBCO^®^).

### Immunohistochemical staining

Details of the procedures used have been described in our previous study [16]. Briefly, 4 μm sections were deparaffinized in xylene and rehydrated in a descending ethanol series. After antigen retrieval in 10 mM sodium citrate (pH 6.0), endogenous tissue peroxidase activities were quenched by 3% hydrogen peroxide for 20 min followed by blocking with 5% normal goat serum (Dako, Glostrup, Denmark) for 1 hour. The sections were immunostained with rabbit monoclonal anti-P4HB (at 1:100 dilution; Cell Signalling Technology Inc., Danvers, MA, USA), rabbit monoclonal anti-PECAM-1/CD31 (1:50 dilution; Abcam^®^, Cambridge, MA, USA), and rabbit monoclonal anti-VEGF antibodies (both at 1:200 dilution; Santa Cruz Biotechnology, Santa Cruz, CA, USA) at 4°C overnight. After incubation with horseradish peroxidase (HRP)-conjugated antibody (Invitrogen-Zymed Laboratories, South San Francisco, CA, USA) at 1:200 dilution, signal was detected using a ready-to-use DAKO EnVision™+ Kit (Dako). Nonspecific immunoglobulin was substituted as negative controls.

### Immunohistochemistry scoring

To evaluate the degree of marker expression in tumour cells, only malignant cells as confirmed by our histopathologist were scored. P4HB and VEGF expressions were assessed semi-quantitatively by the staining intensity (0 = negative, 1 = weak, 2 = moderate, 3 = strong staining) and percentile quadrants of positive cells (0=0%, 1 = 1–25%, 2 = 26–50%, 3=51%–75% and 4 = 75%) in 10 random high-powered fields [45]. A total score varying from 0 to 12 was calculated by multiplying the two measurements. Samples were considered positive if the score exceeded the median value.

Microvessel density (MVD) was evaluated after CD31 staining [46]. Only stained endothelial cells or endothelial cell clusters clearly separated from adjacent microvessels, tumour cells, and connective tissue elements were considered as single, countable microvessel. All slides were initially scanned at low magnification (40× or 100×) for the identification of most prominent vascular ‘hot spots’ followed by MVD counts on five random 200× microscopic fields. The mean value of five counts was recorded and the total score was calculated based on the average of individual counts made by 2 independent observers.

### Immunoblot

25μg of total protein lysates were separated on 12% polyacrylamide SDS gels and electroblotted on nitrocellulose membranes as previously described [47]. Briefly, after blocking with 5% non-fat milk in TBS-T (20 mM Tris, 137 mM NaCl, 0.1% Tween-20, pH 7.6), the membrane was probed with one of the following primary antibodies (at 1:1000 dilution) at 4°C overnight: rabbit monoclonal antibodies against P4HB, total p44/42 MAP kinase (Erk 1/2) (total MAPK), phosphor-p44/42 MAP kinase (Erk1/2) (Thr202/Tyr204) (pMAPK), and VEGF (all from Cell Signaling Technology Inc.). The membranes were washed three times with TBS/T followed by incubation with 1:10,000 diluted HRP-conjugated secondary antibodies (Invitrogen-Zymed Laboratories) at 4°C for 1 hour. Signal on blots was developed using ECL detection system (GE Biosciences, Buckinghamshire, England).

### Semi-quantitative RT-PCR

1 μg of RNA extracts was reverse-transcribed into cDNA using the TaqMan reverse transcription reagents (Applied Biosystems, CA, USA). PCR amplification was performed in a 20 μLreaction mixture containing 3 μLof cDNA template and 200nM of each primer [*P4HB* or beta actin (*ACTB*)]. *ACTB* was used as an internal control for PCR quality. The primer sequences for *P4HB* were 5′-GCTGATGACATCGTGAACTGG-3′ (sense) and 5′- TTGGAGAACACGTCACTGTTG-3′ (antisense) and *ACTB* were 5′-CCAACCGCGAGAAGATGA-3′ (sense) and 5′-CCAGAGGCGTACAGGGATAG-3′ (antisense). The RT-PCR was programmed and started at initial incubation at 94°C for 5 min to activate the Taq DNA polymerase followed by 35 cycles at 94°C for 45 s, 58°C for 45 s, 72°C for 40 s and final extension at 72°C for 10 min. The RT-PCR assay was repeated twice and PCR products were separated by electrophoresis on 2% agarose gel and visualized under UV light after ethidium bromide staining.

### Affymetrix microarray dataset

To interrogate the molecular mechanism underlying P4HB's actions, we studied the gene expression profiles of 73 human specimens from Genomic Spatial Event (GSE) 16011 at the National Center for Biotechnology Information (NCBI) Gene Expression Omnibus (GEO) database (http://www.ncbi.nlm.nih.gov/geo/). These included 7 normal controls, 17 World Health Organization (WHO) grade II, 29 grade III, and 20 grade IV gliomas. The raw CEL files generated from Affymetrix GeneChip Human Genome U133 Plus 2.0 Array were Robust Multi-Array Average (RMA) preprocessed and normalized using GeneSpring software version 12.5 (Agilent Technologies, Santa Clara, CA, USA). The differential gene expression profiles of tissues with low- or high-P4HB expression (using the median expression level as cut-off) were identified. GO enrichment analysis was performed to delineate the predominant functions of up-regulated genes (*p* ≤ 0.00001) under the GO category ‘biological processes’.

### P4HB over-expression, inhibition and subsequent mechanistic in *in vitro* studies

P4HB human cDNA clone was obtained from OriGene Technologies (Rockville, MD, USA). To establish P4HB over-expressed cells, full-length P4HB cDNA flanked with *EcoRI* and *Not I* restriction sites was ligated to pcDNA3.1/V5-His^©^C expression plasmid (Invitrogen™; Life Technologies). pcDNA3.1/V5-His^©^C-P4HB and pcDNA3.1/V5-His^©^C empty vector plasmids were transfected into U87-MG and U251-MG cells using FuGENE^®^6 transfection reagent (Roche Diagnostics, Indianapolis, IN, USA). For further studies, two stable clones with high P4HB expression (P4HB-1 and P4HB-2) and one single clone with empty vector as control (Vec-Ctrl) were selected using neomycin. For *in vivo* imaging, the pLenti-CMV-Puro-LUC lentiviral luciferase vector (Addgene, Cambridge, MA, USA) was transfected into cells for further selection of stably expressed luciferase clones using puromycin. Pharmacologic inhibition of P4HB was achieved with quercetin-3-rutinoside (Q3R) (Sigma-Aldrich) and bacitricin (BAC) (Sigma-Aldrich). MAPK inhibition was achieved by U0126, a small molecule inhibitor (Cell signaling Technology Inc).

### Intracranial xenograft model

Evaluation of tumorigenicity on parental GBM cells and GBM cells with P4HB over-expression was performed using orthotopic tumor model based on guidelines approved by the Committee on the Use of Live Animal for Teaching and Research (CULATR). Immunocompromised [athymic nude (nu/nu)] mice at about six weeks of age were anesthetized by intra-peritoneal injection of 50 mg/kg ketamine/xylazine. Mice were fitted onto a stereotaxic device for intracerebral injections (RWD Life Science Co., Ltd., ShenZhen, GuangDong Province, China). Briefly, a small skin incision was made to expose the bregma suture followed by the creation of a small burr hole at anterior-posterior (AP) = +1 and medial-lateral (ML) = −2.5 from bregma by a micromotor drill (Hager & Meisinger GmbH, Neuss, Germany). 1 × 10^6^ GBM cells in 5μL of PBS (U87-Vec-Ctrl, P4HB-1 and P4HB-2; U251-Vec-Ctrl, P4HB) were slowly deposited (1μL/min) in the right striatum at a depth of −3.5 mm from dura with a 10-μL syringe (26-gauge needle; Hamilton Co., Reno, NV, USA). The needle was left in place for 1 min and slowly withdrawn in 3 min followed by closure of the skin incision with sutures.

### Statistical analysis

All statistical analyses were performed using PASW Statistics 18.0 (SPSS Inc., Chicago, IL, USA). Differentially expressed genes with a fold change ≥ 2 and *p* value ≤ 0.001 were included for Hierarchical Clustering Analysis (HCA) [48] and Gene Ontologies (GO) analysis for the three categories: biological process (BP), molecular function (MF), and cellular component (CC) [49]. Pathway analysis in GeneSpring GX software was used to identify canonical pathways associated with the differentially expressed genes with *p* value ≤ 0.001. Correlation of P4HB expression with different pathological grades were analyzed by Pearson's chi-squared (χ^2^) test. Samples belonging to the P4HB^High^ and P4HB^Low^ groups were defined based on the median value of the sample population. Continuous data were presented as mean ± SD. Student's *t*-test was used to determine whether a difference existed between two groups. One-way ANOVA analysis was used for comparison of more than 2 groups. *P* values of less than 0.05 were considered statistically significant.

## CONCLUSIONS

This study is the first to describe the oncogenic effects of P4HB in glioma and the mechanistic linking of P4HB-mediated MAPK activation in glioma progression, invasion and angiogenesis. It provides new knowledge on the significance of chaperone proteins dysregulation in malignant glioma both clinically and at a molecular level. These findings supplement our previous finding that inhibition of P4HB may resensitise chemoresistant GBM to TMZ. P4HB may be further exploited as a potential predictive marker for GBM prognosis and an alternative therapeutic approach for GBM and possibility in other cancers. Future studies will help to delineation the whole picture of P4HB dependent MAPK signaling together with their molecular interactions in glioma malignancy.

## SUPPLEMENTARY TABLE



## Abbreviations

AP: Anterior-posterior
BAC: Bacitricin
ER: Endoplasmic Reticulum
ERSR: Endoplasmic Reticulum Stress Response
GBM: Glioblastoma Multiforme
GEO: Gene Expression Omnibus
GO: Gene Ontologies
GSE: Genomic Spatial Event
HCA: Hierarchical Clustering Analysis
HGG: High Grade Glioma
LGG: Low Grade Glioma
Q3R: Quercetin-3-Rutinoside
