# Supplementary material for: Endoplasmic reticulum chaperone prolyl 4-hydroxylase, beta polypeptide (P4HB) promotes malignant phenotypes in glioma via MAPK signaling
Source: Oncotarget. 2017 May 19;8(42):71911–23. doi: 10.18632/oncotarget.18026 (PMC5641099; doi:10.18632/oncotarget.18026)
Supplement: Supplementary file 1 [file oncotarget-08-71911-s001.pdf]

# Endoplasmic reticulum chaperone prolyl 4-hydroxylase, beta polypeptide (P4HB) promotes malignant phenotypes in glioma via MAPK signaling

## Supplementary Materials

### Supplementary Table 1: GeneSpring identified canonical signaling pathways that were significantly associated with high P4HB expression (**$p < 0.001$)

| Pathway | *P*-value | Matched Entities | Pathway Entities |
|---|---|---|---|
| Hs_Focal_Adhesion_WP306_41071 | 7.30E-10 | 23 | 188 |
| Hs_Epithelium_TarBase_WP2002_45243 | 9.29E-10 | 22 | 278 |
| Hs_Muscle_cell_TarBase_WP2005_44926 | 9.42E-10 | 24 | 336 |
| Hs_Leukocyte_TarBase_WP2003_44886 | 4.03E-08 | 13 | 128 |
| Hs_Complement_and_Coagulation_Cascades_WP558_45058 | 4.54E-08 | 9 | 64 |
| Hs_Lymphocyte_TarBase_WP2004_46264 | 5.87E-08 | 23 | 420 |
| Hs_BDNF_signaling_pathway_WP2380_54595 | 1.40E-07 | 13 | 141 |
| Hs_EGF-EGFR_Signaling_Pathway_WP437_47973 | 1.52E-07 | 13 | 143 |
| Hs_Regulation_of_Actin_Cytoskeleton_WP51_45278 | 2.11E-07 | 13 | 148 |
| Hs_Squamous_cell_TarBase_WP2006_44276 | 2.25E-07 | 12 | 124 |
| Hs_Inflammatory_Response_Pathway_WP453_41201 | 3.08E-07 | 7 | 33 |
| Hs_TGF_beta_Signaling_Pathway_WP366_47976 | 1.12E-06 | 11 | 118 |
| Hs_DNA_damage_response_(only_ATM_dependent)_WP710_33421 | 4.36E-06 | 9 | 89 |
| Hs_AGE-RAGE_pathway_WP2324_53381 | 4.92E-06 | 8 | 66 |
| Hs_DNA_damage_response_(only_ATM_dependent)_WP710_46091 | 9.11E-06 | 9 | 97 |
| Hs_Integrin-mediated_cell_adhesion_WP185_44862 | 1.39E-05 | 9 | 99 |
| Hs_MAPK_signaling_pathway_WP382_44890 | 2.30E-05 | 11 | 161 |
| Hs_Senescence_and_Autophagy_WP615_47912 | 2.41E-05 | 9 | 106 |
| Hs_Cell_junction_organization_WP1793_44989 | 2.56E-05 | 5 | 28 |
| Hs_Oncostatin_M_Signaling_Pathway_WP2374_54418 | 3.44E-05 | 7 | 63 |
| Hs_Synaptic_Vesicle_Pathway_WP2267_53133 | 9.09E-05 | 6 | 51 |
| Hs_Insulin_Signaling_WP481_42706 | 1.19E-04 | 10 | 160 |
| Hs_Pathogenic_Escherichia_coli_infection_WP2272_53131 | 1.39E-04 | 6 | 64 |
| Hs_Dissolution_of_Fibrin_Clot_WP1802_45190 | 1.58E-04 | 3 | 8 |
| Hs_Integrated_Pancreatic_Cancer_Pathway_WP2377_54420 | 1.64E-04 | 11 | 200 |
| Hs_Hypertrophy_Model_WP516_45364 | 1.72E-04 | 4 | 20 |
| Hs_Wnt_Signaling_Pathway_WP428_43042 | 2.26E-04 | 6 | 61 |
| Hs_RalA_downstream_regulated_genes_WP2290_53118 | 2.34E-04 | 3 | 9 |
| Hs_L1CAM_interactions_WP1843_44884 | 3.04E-04 | 4 | 27 |
| Hs_Endochondral_Ossification_WP474_45000 | 3.23E-04 | 6 | 64 |
| Hs_angiogenesis_overview_WP1993_44954 | 3.23E-04 | 6 | 65 |
| Hs_Calcium_Regulation_in_the_Cardiac_Cell_WP536_44983 | 3.32E-04 | 9 | 149 |
| Hs_IL-6_signaling_pathway_WP364_44627 | 3.35E-04 | 5 | 42 |
| Hs_Spinal_Cord_Injury_WP2431_55909 | 5.93E-04 | 7 | 102 |
| Hs_Oxidative_Stress_WP408_45296 | 8.69E-04 | 4 | 30 |
| Hs_Integrin_cell_surface_interactions_WP1833_44861 | 9.63E-04 | 3 | 16 |
| Hs_Prostaglandin_Synthesis_and_Regulation_WP98_45273 | 9.86E-04 | 4 | 31 |
